# Perinatal depression and maternal-infant bonding disorders in mothers of full-term normal infants

**DOI:** 10.4314/ahs.v26i2.15

**Published:** 2026-06

**Authors:** Tabitha Munyoki, Rose Okoyo Opiyo, Anne Obondo, Felix Ondiek

**Affiliations:** 1 Department of Public and Global Health, Faculty of Health Sciences, University of Nairobi; 2 Department of Psychiatry, Faculty of Health Sciences, University of Nairobi; 3 Department of Food, Nutrition and Dietetics, School of Public Health, Kenyatta University

**Keywords:** Perinatal depression, maternal–infant bonding, postpartum mothers

## Abstract

**Background:**

The period during pregnancy to postnatal is vital for developing of mother–infant bond. A mother suffering from depression may have issues with forming effective bonding. An evaluation of whether perinatal depression influences mother-infant bonding has scarcely been studied in Kenya.

**Objectives:**

This study aimed to evaluate the relationship between perinatal depression and impaired maternal bonding among postpartum mothers.

**Methodology:**

A total of 225 postpartum mothers aged between 18-49 years at 6-12 weeks postpartum at a ratio of 1:2 (cases: controls) were selected. Women who had MIBD were enrolled as cases while mothers without MIBD were controls. Two self-report scale tools; the Postpartum Bonding Questionnaire scale and the Edinburg Postnatal Depression scale were used at 6-12 weeks postpartum to obtain data on depression and bonding. A semi-structured questionnaire collected information on specific risk factors.

**Results:**

The proportion of women with perinatal depression was 40% in the case group and 9.3% in the control group. The odds of having perinatal depression were 6.48 more in the cases than the controls (OR: 6.48, (95% C.I: 3.16, 13.28) and P-value <0.001.

**Conclusion:**

Perinatal depression is associated with impaired maternal bonding at 6-12 weeks postpartum

## Introduction

The World Health Organization (WHO) projected that depression would become a leading source of disease burden in the world by 2020 and beyond^1^. Pregnancy and postnatal period are critical for the development of the maternal-infant bond. Depression is a highly prevalent and diagnosable psychiatric condition. It has been shown to have long-standing consequences that may include affecting the connection between the mother and her infant as well as hampering the child's development.

Maternal–infant bonding refers to the progression of the emotional connection between a mother and her child^2^. These disorders present in different ways, often involving disruptions in this relationship. These include a lack of maternal instinct, bad temper, aggression, or violence as well as neurotic ideas and outright rejection of the infant^3^.

Research generally indicates that perinatal depression and bonding disorders are associated^4^. To avert impaired maternal-infant bonding, depression and other mood disorders must be recognized as soon as possible after delivery, and bonding promptly assessed^5^. Infant mortality in developing countries has remained a major concern despite relative improvement in poverty status and accessibility of contemporary healthcare^6^. Studies on the prevalence of disorders affecting the maternal-infant relationship in sub-Saharan Africa are scarce, though these disorders are treatable and manageable despite their pernicious long-term effects^7^. Studies are needed to explore perinatal depression and its sequela in developing countries.

There is a pressing need to develop integrated care programs aimed at identifying and managing mothers with perinatal depression as well as mother–infant bonding disorders in these settings^8^. An evaluation of whether perinatal depression influences postnatal mother–infant bonding has hardly been researched in Kenya. Given this background, this study aimed to assess whether perinatal depression has any association with maternal-infant bonding at 6–12 weeks postpartum.

## Methodology

### Study design

This was a case–control study, undertaken in a hospital setup within a period of three months, from April to June 2021 among postpartum mothers aged between 18–49 years at 6–12 weeks postpartum.

### Study setting

The study was undertaken in the Mother and Child Health Clinic at Kenyatta National Hospital. The MCH clinic offers primary-level care to patients who reside in the vicinity and also serves as a referral clinic for mother–child pairs who require specialist care.

### Sample Size Determination

Calculations of the sample size were done using the formula for a case–control study (1).


$$n=\left(\frac{r+1}{r}\right)\frac{(\bar{p})(1-\bar{p})(Z_{\beta }+Z_{\alpha /2}{)}^{2}}{(p_{1}-p_{2}{)}^{2}}$$

The following assumptions were considered during the calculation: [i]

- n = sample size per arm
- r = ratio of controls to cases, 1:2 in this case
- P1 = proportion of mothers with depression among those with MIBD, in this case, 29% (2)
- P2 = proportion of mothers without depression among those with MIBD
- P = measure of variability
- Zβ = value corresponding to the power of the study, in this case, 80% = 0.84
- Zα = value corresponding to the normal standard deviate at 95% CI, in this case, = 1.96, with 0.05 level of significance
- P1 – P2 = effect size (difference in proportions)
- Odds ratio to be detected of 2.5 (mother–infant bonding disorders are present at 7%–11.3% in the general population (3), therefore the proportion of mothers with depression among those with MIBD, in this case, 29% (2), and 29% divided by 11.3% gives approximately 2.5). This gave a sample size of 225, where 75 were cases and 150 were controls at a ratio of 1:2.

### Participant's recruitment and screening

The sampling frame was drawn from all mothers attending the mother–child health clinic at Kenyatta National Hospital (KNH), which operates Monday to Friday from 8:00 a.m. to 2:00 p.m. The clinic attends to an average of 40 mothers per day, as well as children of different ages receiving immunization services under the KEPI (Kenya Expanded Programme on Immunization) schedule. The first routine MCH clinic visit is usually scheduled at six weeks postpartum, with a second visit at 10 weeks corresponding to the infant immunization schedule. An additional two-week window was allowed to capture mothers who presented late for their second clinic visit. The study population comprised mothers who delivered at KNH, within Nairobi County, and from other regions of the country, as well as the broader East African region. Participants were women aged 18–49 years who were 6–12 weeks postpartum attending the KNH MCH clinic.

Cases were defined as mothers with postpartum bonding disorders based on the Postpartum Bonding Questionnaire (PBQ), while controls were mothers without bonding disorders. Consecutive sampling was used to recruit cases. For each case enrolled, two controls were recruited from the same source population. Approximately 250 controls were initially identified, and due to the larger pool, simple random sampling was used to select controls at a ratio of 1:2 (75 cases to 150 controls). Two self-report scale tools—the Postpartum Bonding Questionnaire (PBQ) and the Edinburgh Postnatal Depression Scale (EPDS)—were used to obtain data on bonding and depression. Predictor variables included demographic characteristics, obstetric history, partner support, unwanted pregnancy, and intimate partner violence. A semi-structured questionnaire was used to collect obstetric information, partner support, and intimate partner violence data. The antenatal care booklet was used to corroborate selected biological and obstetric variables, including parity, history of pregnancy complications, and age.

### The structured questionnaires

Edinburgh Postnatal Depression Scale (EPDS): This is a 10-item self-report scale used to evaluate the symptomatology of depression. Each item is scored on a four-point scale, with a total score ranging from 0–30. Probable major depression has a cutoff of 12/13, while a cutoff of 9/10 indicates substantial depressive symptoms. Higher EPDS scores indicate a greater likelihood of depression. This scale has been shown to have high internal consistency (Cronbach's alpha = 0.87) and good construct validity^1^. It has been validated for the detection of both forms of maternal depression^2^, and has been widely used in many countries, including Kenya^3^.

**Postpartum Bonding Questionnaire (PBQ):** The Postpartum Bonding Questionnaire was used to assess attachment between mother and child and is one of the most extensively studied tools^1^. The PBQ was developed by Brockington et al.^5^ for the early detection of mother–infant bonding disorders. It consists of four subscales, namely impaired mother–infant bonding, rejection and anger, anxiety about care, and risk of abuse. The items are scored on a 6-point Likert scale ranging from 0 (always) to 5 (never). The PBQ is a valid and sensitive instrument^2^. It can be used in conjunction with the Edinburgh Postnatal Depression Scale (EPDS) for the early identification of mother–infant bonding disorders^2^.

### Statistical methods

Data analysis was conducted using STATA version 11 and results were presented in tables and graphs. Statistical significance was set at p < 0.05. A descriptive analysis of key variables was performed. Bivariate logistic regression analysis was conducted between the binary outcome variable (maternal–infant bonding disorders) and categorical independent variables (maternal sociodemographic characteristics (age, education, marital status,) and obstetric and reproductive health characteristics (parity, delivery mode, pregnancy outcomes, partner support, intimate partner violence and fistula). Variables with borderline or meaningful associations in the bivariate analysis were included in a multivariate logistic regression model to control for confounding and determine independent associations.

## Results

Seventy-five mothers in the case group and 150 in control enrolled and took part in the study which represented 0% attrition (completion rate of 100%).

### Demographic characteristics of mothers of full-term normal infants

The mean age among the cases was 30.2 years (SD = 6.0), compared to 29.1 years (SD = 5.4) among controls. Half (51.4%) of the participants in the cases group were aged 30-44 years followed by 27% aged between 26 and 29 years while majority in the control group were aged 30-44 years (46.0%) followed by 31.2% aged between 18 and 25 years (Table 1). Almost half of the cases had attained tertiary education followed by those with secondary education (45.9%). Among the controls, slightly over half had tertiary education (51.9%) followed by 40.2% with secondary education. With respect to marital status, majority of the cases (90.5%) and controls (89.4%) were married. Nearly equal proportions of majority in among both cases (64.9%) and controls (65.6%) were multiparous. Furthermore, no significant differences existed between the cases and the controls with regard to the four socio-demographic variables (age, education, marital status and parity) analyzed (p>0.05).

**Table 1:** Demographic characteristics of study participants

Maternal-infant bonding disorder				
Variable	Cases, n(%)	Controls, n(%)	OR (95% CI)	p-value
**Age group**				
18-25 y	16(21.6)	59(31.2)	Ref	
26-29 y	20(27.0)	43(22.8)	1.72(0.80-3.69)	0.167
30-44 y	38(51.4)	87(46.0)	1.61(0.82-3.15)	0.164
**Education status**				
Primary school	5(6.8)	15(7.9)	Ref	
Secondary school	34(45.9)	76(40.2)	1.34(0.45-3.99)	0.597
Tertiary education	35(47.3)	98(51.9)	1.07(0.36-3.17)	0.901
**Marital status**				
Married	67(90.5)	169(89.4)	Ref	
Single parent	5(6.8)	15(7.9)	0.84(0.29-2.40)	0.746
Single	2(2.7)	5(2.6)	1.01(0.19-5.33)	0.992
**Parity**				
Multiparous	48(64.9)	124(65.6)	Ref	
Primigravidae	26(35.1)	65(34.4)	1.03(0.59-1.82)	0.909
Mean age	30.2±6.0	29.1±5.4		

### Obstetric and Reproductive Health Characteristics of Participants

The results show that nearly equal proportions of majority among both cases (64.9%) and controls (65.6%) were multiparous (Table 2). Majority of both the cases (61.3%) and the controls (51.3%) had normal vaginal delivery. Among both the cases and the controls, majority (76%) had planned pregnancy. Majority (78% of cases and 84% of controls) indicated that they had a supportive partner while intimate partner violence (IPV) was reported by 16% of cases and 10% of controls. Only 4% of cases and 2% of controls had procured an abortion in the past. Over quarter of the control participants (26.7%) and 20% of the cases had had a miscarriage whereas 4% and 2.7% of the cases and controls respectively reported having suffered a stillbirth. Furthermore, 13.3% and 19.3% of the cases and controls respectively reported a premature birth. The prevalence of fistula was rare in both cases (1.3%) and controls (0.7%). Except for the delivery mode (p=0.024), no significant differences existed between the cases and the controls with respect to their obstetric and reproductive characteristics (p>0.05).

**Table 2:** Obstetric and Reproductive Characteristics of Participants

Variable	Description	Case group (n = 75)	Control group (n = 150)	OR	95% C.I	p-value
**Parity**	Primigravidae	26(34.7%)	55(36.7%)	0.92	(0.51, 1.64)	0.768
	Multiparous	49(65.3%)	95(63.3%)			
**Mode of delivery**	Caesarean section	27(36.0%)	54(36.0%)	Ref		0.024
	Spontaneous Vaginal Delivery	46(61.3%)	77(51.3%)	1.19	(0.66, 2.15)	
	Assisted delivery	2(2.7%)	19(12.7%)	0.21	(0.05, 0.97)	
**Planned pregnancy**	Yes	57(76.0%)	114(76.0%)	Ref	(0.52, 1.91)	1.000
	No	18(24.0%)	36(24.0%)			
**Supportive partner**	Yes	59(78.7%)	126(84.0%)	0.7	(0.35, 1.42)	0.329
	No	16(21.3%)	24(16.0%)			
**IPV**	Yes	12(16.0%)	15(10.0%)	1.71	(0.76, 3.88)	0.2
	No	63(84.0%)	135(90.0%)			
**Procured Abortion**	Yes	3(4.0%)	3(2.0%)	2.04	(0.40, 10.37)	0.394
	No	72(96.0%)	147(98.0%)			
**Miscarriage**	Yes	15(20.0%)	40(26.7%)	0.69	(0.35, 1.35)	0.267
	No	60(80.0%)	110(73.3%)			
**Stillbirth**	Yes	3(4.0%)	4(2.7%)	1.52	(0.33, 6.98)	0.594
	No	72(96.0%)	146(97.3%)			
**Premature birth**	Yes	10(13.3%)	29(19.3%)	0.64	(0.29, 1.40)	0.254
	No	65(86.7%)	121(80.7%)			
**Fistula**	Yes	1(1.3%)	1(0.7%)	2.01	(0.12, 32.64)	0.626
	No	74(98.7%)	149(99.3%)			

### Maternal-infant bonding disorders in mothers of full-term normal infants

**Figure 1:**
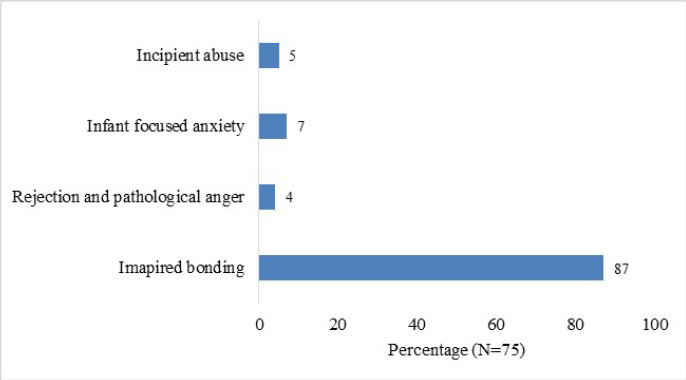
Distribution of MIBD types in the cases of mothers with full-term normal infants in KNH

Figure 1 illustrates that majority of cases had impaired bonding (87%) while the remaining 7%, 5% and 4% of the cases had infant-focused anxiety, incipient abuse and rejection and pathological anger respectively. Two cases had multiple categories of bonding disorder (i.e., both infant-focused anxiety and impaired bonding).

### Prevalence of depression among cases and controls

**Figure 2:**
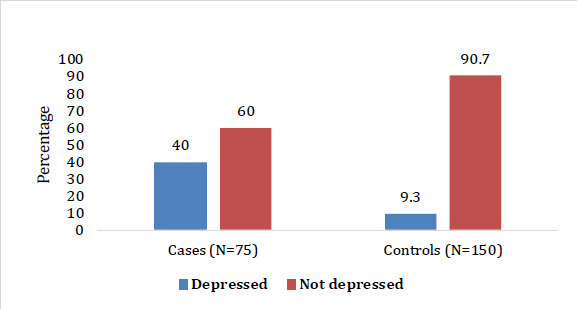
Prevalence of depression among cases and controls

Forty percent of cases had perinatal depression, while 60% did not. A minority, 9.3% of controls had depression, whereas 90.7% did not (Figure 2).

**Figure 3:**
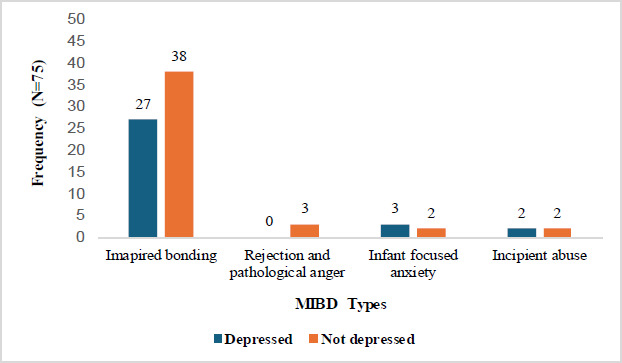
Distribution MIBD types by maternal depression status (N=75)

Twenty-seven cases with impaired bonding suffered depression while 38 did not. All cases that experienced rejection and pathological anger were depressed. Three of the five cases with infant focused anxiety suffered depression while half of the 4 cases that experienced incipient abuse were depressed (Fig 3).

### Factors Associated with Maternal-Infant Bonding Disorders among Mothers of full-term Normal Infants

#### Bivariate Analysis

As shown in Table 3, Association between sociodemographic, obstetric and reproductive health characteristics, and the occurrence of maternal-infant bonding disorders (MIBD) was tested using bivariate logistic regression analysis. None of the socio-demographic variables (age, education and marital status) shown were significantly associated with having MIBD (i.e., being in the case or control group) (p>0.05).

**Table 3:** Factors associated with maternal-infant bonding disorders in mothers of full-term normal

Variable	Description	Case group (n = 75)	Control group (n = 150)	OR	95% C.I	p-value
**Age**	18-25 y	16(21.3%)	46(30.7%)	Ref		0.318
	26-29 y	20(26.7%)	37(24.7%)	1.55	(0.71, 3.41)	
	30-44 y	39(52.0%)	67(44.7%)	1.67	(0.84, 3.34)	
**Education**	Primary	5(6.7%)	14(9.3%)	Ref		0.291
	Secondary	35(46.7%)	54(36.0%)	1.81	(0.60, 5.49)	
	Tertiary	35(46.7%)	82(54.7%)	1.2	(0.40, 3.57)	
**Marital status**	Married	68(90.7%)	131(87.3%)	Ref		0.75
	Separated	5(6.7%)	14(9.3%)	0.69	(0.24, 1.99)	
	Single	2(2.7%)	5(3.3%)	0.77	(0.15, 4.08)	
**Parity**	Primigravidae	26(34.7%)	55(36.7%)	0.92	(0.51, 1.64)	0.768
	Multiparous	49(65.3%)	95(63.3%)			
**Mode of delivery**	Caesarean section	27(36.0%)	54(36.0%)	Ref		0.024
	Spontaneous Vaginal Delivery	46(61.3%)	77(51.3%)	1.19	(0.66, 2.15)	
	Assisted delivery	2(2.7%)	19(12.7%)	0.21	(0.05, 0.97)	
**Planned pregnancy**	Yes	57(76.0%)	114(76.0%)	Ref	(0.52, 1.91)	1.000
	No	18(24.0%)	36(24.0%)			
**Supportive partner**	Yes	59(78.7%)	126(84.0%)	0.7	(0.35, 1.42)	0.329
	No	16(21.3%)	24(16.0%)			
**IPV**	Yes	12(16.0%)	15(10.0%)	1.71	(0.76, 3.88)	0.2
	No	63(84.0%)	135(90.0%)			
**Procured Abortion**	Yes	3(4.0%)	3(2.0%)	2.04	(0.40, 10.37)	0.394
	No	72(96.0%)	147(98.0%)			
**Miscarriage**	Yes	15(20.0%)	40(26.7%)	0.69	(0.35, 1.35)	0.267
	No	60(80.0%)	110(73.3%)			
**Stillbirth**	Yes	3(4.0%)	4(2.7%)	1.52	(0.33, 6.98)	0.594
	No	72(96.0%)	146(97.3%)			
**Premature birth**	Yes	10(13.3%)	29(19.3%)	0.64	(0.29, 1.40)	0.254
	No	65(86.7%)	121(80.7%)			
**Fistula**	Yes	1(1.3%)	1(0.7%)	2.01	(0.12, 32.64)	0.626
	No	74(98.7%)	149(99.3%)			

Among the obstetric and reproductive health variables, only assisted delivery showed a significant association with MIBD (being in the case group): Compared to Caesarean section (reference), assisted delivery was associated with 79% lower odds of being having MIBD (OR = 0.21; 95% CI: 0.05–0.97). Women who had spontaneous vaginal delivery showed slightly higher odds of having MIBD compared to their peers who went thorough caesarean section, however this was not statistically significant (OR = 1.19; 95% CI: 0.66–2.15).

#### Multivariate Model

All variables that had a threshold of p < 0.20 in the bivariate analysis were entered in the multivariate analysis model. Maternal age was included a priori, despite not meeting this threshold because it is a known determinant of health exposures and outcomes and could confound relationships between mode of delivery and MIBD. The results show that Perinatal depression was the only independent, statistically significant predictor of being MIBD: Women with perinatal depression had over 7 times higher odds of having MIBD relative to those without depression (aOR = 7.30; 95% CI: 3.30–16.13; p < 0.001). Furthermore, perinatal depression showed a strong effect on the outcome (MIBD) as indicated by the large effect size (aOR = 7.30) and narrow confidence interval (3.30–16.13). This presents strong evidence against the null hypothesis and a significant association between perinatal depression and impaired maternal–infant bonding at 6–12 weeks postpartum. The Mode of delivery – particularly assisted delivery – showed a borderline, but not significant, association (p = 0.06) with MIBD. The odds of having MIBD among women undergoing assisted delivery was reduced by 80% (aOR = 0.20) compared to their counterparts undergoing C-section. While not reaching conventional statistical significance (p < 0.05), the magnitude is notable and may warrant consideration in larger studies or as a trend.

**Table 4:** Predictors of maternal-infant bonding disorders in mothers of full-term normal infants

Variable	Description	Case group (n = 75)	Control group (n = 150)	AOR	95% C.I	P- value
**Age**	18-25 y	16(21.3%)	46(30.7%)	1		
	26-29 y	20(26.7%)	37(24.7%)	1.34	(0.56, 3.23)	0.511
	30-44 y	39(52.0%)	67(44.7%)	1.43	(0.66, 3.11)	0.358
**Mode of delivery**	CS	27(36.0%)	54(36.0%)	1		
	SVD	46(61.3%)	77(51.3%)	1.60	(0.81, 3.13)	0.173
	Assisted	2(2.7%)	19(12.7%)	0.20	(0.04, 1.07)	0.06
	delivery					
**Supportive partner**	Yes	59(78.7%)	126(84.0%)	0.89	(0.41, 1.95)	0.778
	No	16(21.3%)	24(16.0%)	1		
**IPV**	Yes	12(16.0%)	15(10.0%)	1.00	(0.38, 2.68)	0.992
	No	63(84.0%)	135(90.0%)	1		
**Perinatal depression**	Yes	30(40.0%)	14(9.3%)	7.30	(3.30, 16.13)	<0.001
	No	45(60.0%)	136(90.7%)	1		

## Discussion

This study did not establish any significant associations between the socio-demographic variables (maternal age, education, marital status, or parity) and MIBD – which is contrary to much of existing literature^9^-^11^. A study in Sweden showed that younger mothers had a higher risk of reporting, suppressing, and expressing problems in their children^1^ likely due higher rates of unplanned pregnancies and lower rates of psychological maturity among them^2^. Conversely, other studies have shown that older women have a higher probability of developing depression during pregnancy and may experience challenges bonding with their infants^2^. This could be attributable to distinct challenges that they face including pregnancy complications, obstetrical interventions and fatigue, all of which would impair bonding^12^. The lack of an age effect in current findings is partly attributable to the relatively small sample size within each age stratum, which limited statistical power to detect modest effects.

Current findings on the association between marital status and MIBD are consistent with those of Afolabi et al^1^, which indicated that being single is not necessarily associated with developmental problems in children. However, in African settings, cultural stigma surrounding single parenthood may predispose mothers to depression and bonding difficulties^3^. In these contexts, single parenting is often associated with reduced social support which compounds to maternal stress and eventually degenerates to maternal depression and bonding challenges. Although general evidence indicates that higher education is associated with lower rates of depression^4^, current findings found no statistically significant association despite tertiary education showing slightly lower odds of MIBD development. This was likely due to fact that most participants (> 90%) had secondary to tertiary education resulting in limited variability. Higher education is generally protective against depression, and by extension, MIBD, potentially via improved health literacy, economic opportunities, and access to resources^13^.

A majority of participants reported adequate social support, and few reported intimate partner violence (IPV), which may explain the lack of association observed. This contrasts with studies showing that depression is associated with lack of social support, single parenthood, IPV, poverty, younger age, and unplanned pregnancy^6^. Furthermore, IPV during pregnancy has been associated with mother–infant bonding difficulties postpartum5. The low reporting of IPV and lack of partner support in this study may be due to stigma and limited awareness of gender-based violence, leading to underreporting.

Selected obstetric characteristics, including parity, mode of delivery, and obstetric complications, were not significantly associated with MIBD. However, other studies have identified stillbirth, difficult delivery, and unplanned pregnancy as risk factors for bonding disorders^14^,^15^. The lack of significant findings in this study may be explained by the small proportion of participants reporting assisted delivery or obstetric complications. Larger studies may provide more accurate estimates of these associations.

The prevalence of perinatal depression among controls was 9.3%, which is comparable to previous findings reporting 8.5–10% antenatal and 6.5–12.9% postnatal depression rates^8^. Higher prevalence rates have been reported in sub-Saharan Africa, including 26.6% in Ghana and 32.9% in Côte d'Ivoire^16^, while studies in East Africa report rates of approximately 20%^8^. Depression may impair maternal adjustment and responsiveness to infant cues, resulting in suboptimal bonding^13^. Even mild depressive symptoms have been shown to negatively affect bonding and child development^17^.

It is worth noting that among cases with established bonding disorders, the prevalence of depression rose to 40%, a four-fold increase. This gradient suggests that while depression may not be present in the majority of postpartum women, its co-occurrence with bonding difficulties is substantially elevated. The high prevalence of maternal depression among cases with MIBD is consistent with previous findings, where approximately 29% of mothers with perinatal depression exhibit bonding disorders^18^. Studies in India have also reported higher rates of bonding disorders among mothers with psychiatric illness compared to those without^19^. In the general population, bonding disorders affect approximately 7–11.3% of mother–infant pairs^20^, which corroborates with current finding of 9.3% among the control group.

In this study, mothers with perinatal depression had over 7 times higher odds of having MIBD than their peers without depression. This finding aligns with a growing body of international evidence linking maternal mood disorders to disruptions in the early mother–infant relationship: Previous studies have identified perinatal depression as a key independent predictor of bonding disorders^21^–^23^. Impaired bonding has been associated with increased risk of abusive parenting and adverse child outcomes^24^–^26^. The strong association between perinatal depression and MIBD provokes important questions about the underlying mechanism: Depressed mothers often exhibit flattened or negative affections, reduced responsiveness to child cues, less frequent and reduced quality of both verbal and non-verbal communication and emotional volatility. Such behavioral patterns militate against the establishment of attachment between mother and child^27^–^29^. There remains debate regarding the direction of this relationship—whether maternal depression leads to bonding disorders or vice versa 4,26,30. While the prevailing view is that depression precedes and causes bonding impairment, some evidence suggests that poor bonding can itself, in certain circumstances, precipitate or exacerbate maternal depression 24,27,31.

Due to the case–control design, this study could not establish temporal causality between maternal depression and bonding disorders since both were assessed concurrently at 6-12 weeks postpartum. Longitudinal research that assesses postpartum depression and maternal-infant bonding repeatedly from antenatal period through one year postnatal may be necessary to establish temporal precedence and identify critical windows for intervention.

## Conclusion and Recommendation

This study provides evidence that perinatal depression is strongly and independently associated with impaired maternal–infant bonding at 6–12 weeks postpartum, in Kenya. Women with depression had seven-fold higher odds of bonding disorders compared to their non-depressed peers About half of postpartum mothers with impaired bonding had perinatal depression. Most women with bonding disorders had impaired bonding compared to the other three types of bonding disorders.

This study found that none of the sociodemographic, obstetric, or reproductive health factors examined—in-cluding age, education, marital status, parity, delivery mode, partner support, or IPV were significantly associated with maternal–infant bonding disorders. The study recommends early perinatal mental/psychological health screening: to identify and offer prompt treatment to mothers showing signs of postpartum depression, anxiety, or trauma since they are strongly linked to impaired bonding. The high prevalence of impaired bonding among the MIBD types calls for intensified promotion of postpartum practices that enhance mother-child bonding including skin-to-skin contact, early initiation of breastfeeding (EIBF), and other breastfeeding support, and responsive caregiving. More comprehensive research (longitudinal studies) is needed to monitor bonding outcomes from pregnancy through early childhood to understand multidimensional causes and long-term effects. Overall, the findings underscore the importance of early identification and support for mother–infant pairs in the early postpartum period, which is critical for the development of the mother–child bonding relationship.
